# Elemental Profile, General Phytochemical Composition and Bioaccumulation Abilities of Selected *Allium* Species Biofortified with Selenium under Open Field Conditions

**DOI:** 10.3390/plants12020349

**Published:** 2023-01-11

**Authors:** Sandra Vuković, Djordje Moravčević, Jelica Gvozdanović-Varga, Biljana Dojčinović, Ana Vujošević, Ilinka Pećinar, Sofija Kilibarda, Aleksandar Ž. Kostić

**Affiliations:** 1 Faculty of Agriculture, University of Belgrade, Nemanjina 6, 11080 Belgrade, Serbia; 2 Institute of Field and Vegetable Crops, Maksima Gorkog 30, 21000 Novi Sad, Serbia; 3 Institute for Chemistry, Technology and Metallurgy, National Institute of Republic of Serbia, University of Belgrade, Njegoševa 12, 11000 Belgrade, Serbia

**Keywords:** alliums, biofortification, biogenic elements, secondary metabolites, selenium

## Abstract

*Allium* species are known as a rich source of many compounds with potential healing effects. Biofortification is recognized as an effective agrotechnical measure for raising the level of biogenic elements—especially microelements in the edible parts of these species, so *Allium* can be considered as a ‘natural dietary supplement’. The aim of this research was to test the effects of foliar application of Se fertilizer (Na_2_SeO_4_) in different doses (control—0, 10, 20 and 30 g per ha) on the content of macro, microelements and secondary metabolites (SMs)—free phenolics, flavonoids and hydroxycinnamic acid derivatives in the edible parts, i.e., leaves of two selected *Allium* species in Serbia (*A. odorum* and *A. schoenoprasum*), which grew in open field conditions over the course of two growing seasons. The bioaccumulation factor (BAF), as an indicator of the ability of plants to accumulate biogenic elements, was also determined. Although with no full regularity, the dose of 10 g of Se per ha yielded the highest content for the most biogenic elements for both alliums in the first growing season, i.e., a dose of 20 g of Se per ha for *A. schoenoprasum*, and a dose of 30 g of Se per ha for *A. odorum* in the second growing season. The obtained results justified the Se-biofortification of different alliums. The BAF values indicated the ability of both *Allium* species to accumulate S, K and P in their leaves during both growing seasons. The accumulation of potentially toxic elements was not recorded for either species, emphasizing the safety of the produced plant material for human consumption. Additionally, Se-treated plants had higher SM contents compared to control plants. The growing season also showed an impact on SM content; i.e., in the second season, characterized as drought-stressed, the synthesis of SMs was significantly higher compared to that in the first season. Further research should be directed towards finding the appropriate dose of Se, expanded in the sense of conducting research in controlled conditions, as well as different ways of applying Se fertilizer. The idea of this study was also to popularize the examined *Allium* species, which are rarely grown in the territory of Serbia.

## 1. Introduction

The genus *Allium* belongs to the family Amaryllidaceae, and it is one of the largest genera of monocotyledonous plants. According to a newer classification, it is divided into 15 subgenera with 72 sections, and it includes more than 800 species distributed throughout the Northern hemisphere [1]. 

*Allium* species have been known for centuries for their phytotherapeutic effects. Today, their application is widespread, and they are used as a remedy in folk medicine and for vegetables, spices or flowers. In many countries, especially in Asia, these species are also economically important crops [2]. The most commonly cultivated *Allium* species are *A. sativum* L. (garlic), *A. cepa* L. (onion), *A. porrum* L. (leek), *A. ascalonicum* L. (shallot), *A. fistulosum* L. (scallion), *A. schoenoprasum* L. (chives), etc. [3]. Other, lesser-known *Allium* species such as *A. odorum* L., *A. nutans* L. and *A. ascalonicum* L. are mostly grown locally in home gardens. 

Depending on the morphology or the degree of maturity of the plants, their usually edible parts are bulbs, leaves, flowers [4] or whole plants—known as spring onions. 

Considering the chemical composition, *Allium* species contain the same compounds but at different levels [5]. Major constituents of *Allium* species are sulphur-containing compounds, i.e., S-alk(en)yl-L-cysteine sulfoxides and their precursors. Some authors indicate that the phytotherapeutic effects, flavor and quality of alliums are largely determined by the presence of these compounds [6,7,8]. Further, alliums are also a rich source of secondary metabolites (SMs) such as phenolics (in particular flavonoids), phytosterols and saponins, which exhibit numerous biological activities [2]. The most abundant flavonoid is quercetin, followed by anthocyanin pigments. Flavonoids are known as antioxidant agents, while the roles of phytosterols and saponins are still not fully clarified [9]. The synthesis and accumulation of SMs mainly depend on environmental conditions [10]. Namely, plants under harmful environmental conditions increase the synthesis of SMs in order to provide resistance from the negative consequences of abiotic stresses, such as oxidative stress, cell membrane peroxidation, etc. [11,12]. Considering that SMs are recognized as health-related compounds, their increased content in plant-based food also indicates their higher biological value [13]. On the other hand, in order to obtain an accurate phytochemical profile, it is recommended to grow plants in carefully controlled conditions [10].

The nutritional value of *Allium* species is determined by the presence of carbohydrates, proteins, fiber, ash, lipids and vitamins (especially vitamin C and E and B-complex vitamins), as well as biogenic elements.

According to various authors, from 16 to 25 biogenic elements, characterized as macro and microelements, are recognized as essential for plants, animals and humans. Macroelements include nitrogen, phosphorus, potassium, calcium, magnesium and sulphur [14,15,16].

Microelements include zinc, iron, manganese, copper, molybdenum, boron, chlorine and nickel as essential, and silicon, sodium, cobalt and strontium as beneficial for plants. In addition, selenium, iodine and chromium are essential for humans and animals, while their essentiality for plants has not been fully elucidated [14,15,16]. 

Since the human body cannot synthesize biogenic elements, special attention is paid to mineral nutrition of plants, with the aim of producing food with high nutritional value [14,15,16]. 

*Allium* species are recognized as a good source of some important biogenic elements, and they are appreciated as functional food that contributes to overall health and disease prevention. Most authors indicate a medium level of Mg, P and Fe and a high content of K, while some authors also point out the high amount of Se in their edible parts [3,8,17]. 

Agrofortification or agronomic biofortification as a segment of biofortification is a cost-effective and sustainable method based on the enrichment of edible parts of plants by applying appropriate mineral nutrition and agro-technical measures. Biofortification implies the application of fertilizers with microelements by introducing them into the soil and using foliar or seed treatment [18,19].

Selenium (Se) is an essential element for humans, and its deficiency leads to many diseases, such as cardiovascular disease, infertility and mental and musculoskeletal disorders [20]. On the other hand, Se is not essential for plants, with some exceptions, but the results of some research indicate that its application has multiple benefits for plants; it reduces the negative effect of various abiotic stresses, and it reduces bud abortion [21,22]. 

Hence, the present study was carried out with the aim to determine the content of macro and microelements in edible parts, specifically, leaves of two selected *Allium* species (*A. odorum* and *A. schoenoprasum*), which were grown in open field conditions and biofortified with different doses of Se fertilizer, via inductively coupled plasma–optical emission spectrometry (ICP-OES). The bioaccumulation factor (BAF) of macro and microelements was also calculated in order to estimate the potential of *Allium* species to accumulate primarily Se, as well as other biogenic elements. In addition, considering the importance of plant secondary metabolites, the total content of free phenolics (TPC), flavonoids (TFC) and hydroxycinnamic acid derivatives (HCAs) was also determined, with the aim of evaluating the potential health value of edible parts of selected biofortified alliums. 

## 2. Results

### 2.1. Soil Analysis

Table 1 shows the contents of macro and microelements in the soil in the experimental field, which were analyzed via ICP-OES with suggested ranges according to the available literature included in Table 1. The content of all tested elements was expressed as mg kg^−1^ of air-dried soil.

The contents of biogenic elements in the soil were determined by several factors: parent material from which the soil was formed, and pedogenetic and anthropogenic processes (fertilization, pollution). Each of the elements exists in the soil in its specific form and shows different availability to plants. The obtained results indicated that the supply of the tested soil with biogenic elements was within the world average [23,25,29]. Furthermore, the content of potentially toxic elements (Pb, Cd, Cr, Ba, As) was below the maximum allowed level prescribed by the US Environmental Protection Agency (EPA) [24]. In addition, the content of Se was below the detection limit of the instrument (<1.0 µg g^−1^), which indicates low supply of soil with this element and justifies its application in the biofortification process with the aim of enriching selected *Allium* species.

### 2.2. Analyses of Plant Material 

The contents of macro (Ca, K, P, Mg, S) and microelements (Al, As, B, Ba, Cd, Co, Cr, Cu, Fe, Li, Mn, Na, Ni, Pb, Se, Sr, Zn) measured in edible parts (leaves) of *A. odorum* and *A. schoenoprasum* for both seasons was analyzed via ICP-OES. The content of all tested elements was expressed as µg g^−1^ of fresh weight (FW). The BAF values, as indicators of the ability of selected *Allium* species to accumulate tested elements, for both seasons, were also calculated. Further, the general phytochemical composition—the total content of phenolics (TPC), flavonoids (TFC) and hydroxycinnamic acid derivatives (HCAs)—in edible parts of analyzed *Allium* species, for both seasons, was determined. The obtained results were expressed as mg of the corresponding equivalents per g of fresh weight (FW).

#### 2.2.1. *A. odorum*—Elemental Profile (Season I)

The contents of biogenic elements observed in the leaves of *A. odorum* are presented in Table 2. Namely, during the first season, in the sequence of macroelements, findings showed that the lowest content of Ca was in the control condition (without application of Se fertilizer) and the highest in the treatment with 10 g of Se fertilizer per ha. The application of Se in doses of 20 and 30 g per ha statistically significantly reduced the concentration of Ca in the leaves compared to the dose of 10 g per ha. Contrastingly, the lowest contents of K, Mg and P were observed in the treatment with 10 g of Se per ha, while the highest values were determined in the treatment with 20 g of Se per ha for K and P, and in the treatment with 30 g of Se per ha for Mg. Observing the content of S, the highest value was measured in the control condition, while in the treatments with 10, 20 and 30 g per ha, the content was statistically significantly lower compared to the control. 

Considering the content of microelements, the results showed that in the case of B, there was a statistically significant difference between all treatments, with the lowest value recorded in the treatment with 20 g of Se per ha and the highest in the treatment with 10 g of Se per ha. Concerning Cu, the control condition and the treatment with 10 g of Se per ha showed statistically significantly higher values compared to the treatments with 20 and 30 g of Se per ha. The highest Fe content was measured in the treatment with 10 g of Se per ha, while the lowest was observed in the control condition, which was not statistically significantly different compared to the content measured in the treatment with 30 g of Se per ha. The highest content of Mn was recorded in the control condition, while among the treatments with Se fertilizer (10, 20, 30 g per ha), the content did not significantly statistically differ. The Na content ranged between 14.11 µg g^−1^ (FW) and 21.42 µg g^−1^ (FW), which corresponded to the application of 20 and 30 g of Se per ha, respectively. Application of Se at 10 g per ha did not statistically significantly affect the Na content compared to the control condition. The lowest Zn content was observed for 10 g of Se per ha and the highest for 20 g of Se per ha, which did not statistically significantly differ compared to the control condition. 

As was expected, the application of Se fertilizer statistically significantly increased the Se content in the leaves of *A. odorum* in all tested treatments.

#### 2.2.2. *A. odorum*—Elemental Profile (Season II)

The contents of macro and microelements in the leaves of *A. odorum* in the second season are also shown in Table 2. The results indicate that the lowest contents of Ca and Mg were measured in the control condition, which did not statistically significantly differ compared to the content observed in the treatment with 10 g of Se per ha, for both tested elements. The highest contents of Ca and Mg were recorded in the treatments with 30 and 20 g of Se per ha, respectively. Regarding K, the content in all tested treatments was statistically significantly different; the lowest content corresponded to 30 g of Se per ha, and the highest corresponded to the control condition. The highest dose of Se fertilizer (30 g per ha) statistically significantly increased the content of P compared to other treatments (control, 10 and 20 g Se per ha). In the treatment with 20 g of Se per, ha the lowest content of P was measured, which did not statistically significantly differ compared to the control condition. The highest content of S was recorded in the treatment with 20 g of Se per ha, and the lowest was observed in the treatment with 30 g of Se per ha, but the obtained content did not statistically significantly differ compared to the content in the control condition and treatment with 10 g of Se per ha.

In the sequence of microelements, results indicated that the lowest content of B was observed in the treatment with 20 g of Se per ha and the highest in the treatment with application of 30 g of Se fertilizer per ha. In addition, for Cu, the highest content was measured in the treatment with 30 g of Se per ha, but for both elements (B and Cu), the observed contents in the control condition and in the treatment with 10 g of Se per ha did not statistically significantly differ. In the case of Fe, with an increased dose of Se fertilizer, the content of Fe also increased, so the lowest content was recorded in the control condition and the highest in the treatment with 30 g of Se per ha. The content of Mn in the control condition and in the treatment with 10 g of Se per ha was statistically significantly lower compared to the content observed in treatments with 20 and 30 g of Se per ha. For Na, in the control condition, the presence of this element was not determined, while the other treatments statistically significantly differed from each other; the lowest content was measured in the treatment with 10 g of Se per ha, and the highest was observed in the treatment with 20 g of Se per ha. When it comes to Zn, lower contents were recorded in the control condition and the 20 g per ha treatment, among which there was no statistically significant difference. Higher contents of Zn were observed in the treatment with 10 and 30 g per ha, and between these treatments as well, there was no statistically significant difference.

The application of Se fertilizer had a statistically significant effect on the increase of Se content in the edible parts (leaves) of *A. odorum*; the lowest content was determined in the control condition and the highest in the treatment with 30 g of Se per ha.

#### 2.2.3. Bioaccumulation Factor (BAF)—*A. odorum*

Table 3 shows the values of the bioaccumulation factor for *A. odorum* for both seasons. The BAF values for K and S, for both seasons and in all applied treatments, were greater than one. For P, BAF values for all treatments were between 0.5 and 1. For Ca, the BAF values ranged between 0.35 and 0.56 for the first season and from 0.28 to 0.37 for the second season. In addition, in the first season, BAF values for B were between 0.0 and 0.5, while in the second season, their BAF values were 0.0. For other elements, BAF values were also 0.0 in both seasons. Furthermore, what is also extremely important is that none of the potentially toxic elements was accumulated. 

#### 2.2.4. Phytochemical Composition (TPC, TFC, HCAs) of *A. odorum* (Both Seasons)

**Figure 1:**
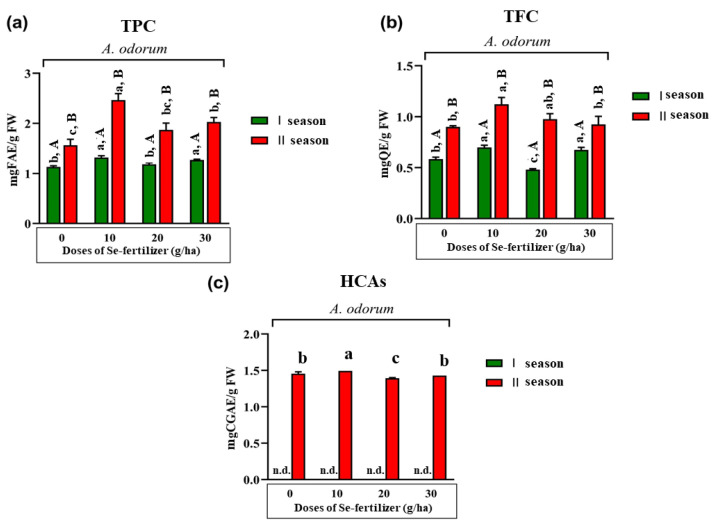
Effect of different doses (control—0, 10, 20 and 30 g per ha) of Se fertilizer and growing season on the total content of free phenolics (TPC): (**a**) total content of free flavonoids (TFC); (**b**) and total content of free hydroxycinnamic acid derivatives (HCAs) (**c**) in the edible parts (leaves) of *A. odorum*. The bars with (+) standard deviation represent mean values; lowercase letters indicate comparisons between treatments (applied doses of Se-fertilizer (g/ha)); uppercase letters indicate comparisons between seasons; different letters indicate statistically significant differences according to Tukey’s HSD test (*p* < 0.05). n.d.—not detected; FAE—ferulic acid equivalents; QE—quercetin equivalents; CGAE—chlorogenic acid equivalents.

Figure 1a–c show TPC, TFC and HCAs in the edible parts (leaves) of *A. odorum* for both seasons.

Significantly higher TPC, TFC and HCAs were observed in the second growing season compared to the first one.

In the first season, the application of 10 and 30 g of Se fertilizer per ha provoked statistically higher TPC and TFC compared to the control and treatment with 20 Se g per ha. In the second season, the highest TPC was achieved in the treatment with 10 g of Se per ha, which was statistically significantly higher than the control and other treatments (20 and 30 g Se per ha). In addition, in the second season, the highest TFC was measured in the treatment with 10 g of Se fertilizer per ha but was not statistically significantly different compared to the content achieved in the treatment with 20 g of Se per ha. In the case of HCAs, during the first growing season, these derivatives of phenolic acids were not detected, while in the second season in the treatment with 10 g Se per ha, statistically higher content was recorded compared to the content measured in other treatments (control, 20 and 30 g of Se per ha).

#### 2.2.5. *A. schoenoprasum*—Elemental Profile (Season I)

The content of biogenic elements (macro and micro elements) measured in the leaves of *A. schoenoprasum* in both seasons is presented in Table 4.

Considering the content of macroelements, a statistically significant difference for Ca was observed between the treatment with 10 g of Se per ha, as the highest value, and the treatment with 20 g of Se per ha, as the lowest value. In contrast, the highest content of K was measured in the treatment with 20 g of Se per ha and the lowest in the treatment with 10 g per ha; however, the achieved content in all treatments did not statistically significantly differ. In the case of Mg, the highest content was observed in the treatment with 10 g of Se per ha, which was statistically significantly different compared to the content observed in the treatment with the maximum dose of Se fertilizer (30 g per ha), as the lowest. Moreover, a statistically significantly higher content of P was recorded in the treatment with 10 g of Se per ha compared to the content observed in other treatments (control, 20 and 30 g Se per ha).

Concerning S, higher doses of Se fertilizer (20 and 30 g per ha) statistically significantly increased its content compared to the content obtained in the control condition and the treatment with 10 g of Se per ha.

In the sequence of microelements, the contents of B and Zn statistically significantly increased in the treatment with 10 g per ha of Se fertilizer, compared to other treatments (control, 20 and 30 g Se per ha). When it comes to Cu and Fe, the highest contents were observed in the treatment with 20 g of Se per ha, while the lowest contents of both elements were measured in the treatment with the highest dose of Se (30 g per ha). The doses of 10 and 30 g of Se fertilizer statistically significantly decreased the content of Na compared to the control condition and treatment with 20 g Se per ha. 

The content of Se, as in *A. odorum*, statistically significantly increased with increases in the dose of Se fertilizer.

#### 2.2.6. *A. schoenoprasum*—Elemental Profile (Season II)

The contents of biogenic elements in the leaves of *A. schoenoprasum* in the second season is shown in Table 4. 

The application of Se fertilizer in doses of 10 and 30 g per ha statistically significantly decreased the content of Ca compared to the content observed in the control condition and the treatment with 20 g of Se per ha. K and Mg in the control condition exhibited statistically significantly higher contents compared to the contents measured in treatments with Se fertilizer (10, 20 and 30 g Se per ha). For P, the highest content was observed in the treatment with a maximum dose of Se fertilizer (30 g per ha), but it was not statistically significantly higher compared to the content that was observed in the control condition. The lowest content of P was measured in the treatment with 20 g of Se per ha. Using higher doses of Se fertilizer (20 and 30 g per ha) contributed to a statistically significant increase in the content of S compared to the control condition and treatment with 10 g of Se per ha.

In the microelement sequence, the highest contents of B, Cu, Mn and Na were measured in the control condition, but for Cu and Mn, the contents did not statistically significantly differ compared to the contents observed in the treatments with 30 g of Se per ha. The lowest content of the mentioned elements was observed in the treatment with 10 g of Se per ha. The dose of 20 g Se fertilizer per ha statistically significantly increased the content of Fe compared to the content observed in other treatments. The highest content of Zn was observed in the treatment with 30 g of Se per ha and the lowest in the treatment with 10 g of Se per ha. 

As in the previous cases, applying Se fertilizer statistically significantly increased the content of this element in the leaves of *A. schoenoprasum*.

#### 2.2.7. Bioaccumulation Factor (BAF)—*A. schoenoprasum*

The values of the bioaccumulation factor for *A. schoenoprasum*, for both seasons, are presented in Table 5. The results showed that the accumulation of tested elements in the first season was more pronounced compared to the second season.

In particular, BAF values greater than one were recorded only for S in the first season, while in the second season, the BAF values for S ranged between 0.7 and 0.8. Furthermore, the BAF values for K in both seasons and the BAF values for P in the first season were lower than 1 (ranged between 0.5 and 0.9), while the BAF values for P in the second season were below 0.5 (about 0.3). The accumulation of other elements was not prominent; i.e., the BAF values were 0.1 or lower, in both seasons. This *Allium* species also did not accumulate potentially toxic elements in the edible parts, in both seasons.

#### 2.2.8. Phytochemical Composition (TPC, TFC, HCAs) of *A. schoenoprasum* (Both Seasons)

**Figure 2:**
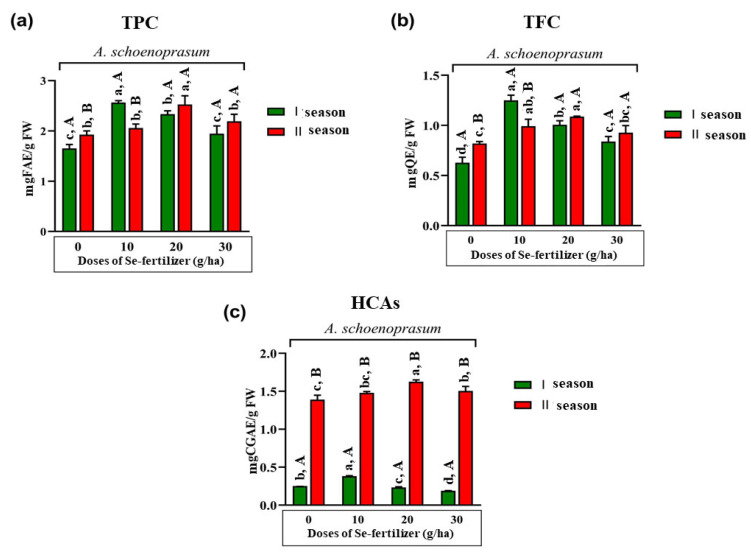
Effect of different doses (control—0, 10, 20 and 30 g per ha) of Se fertilizer and growing season on the total content of free phenolics (TPC) (**a**), total content of free flavonoids (TFC) (**b**) and total content of free hydroxycinnamic acid derivatives (HCAs) (**c**) in the edible parts (leaves) of *A. schoenoprasum*. The bars with (+) standard deviation represent mean values; lowercase letters indicate comparisons between treatments (applied doses of Se-fertilizer (g/ha)); uppercase letters indicate comparisons between seasons; different letters indicate statistically significant differences according to Tukey’s HSD test (*p* < 0.05). n.d.—not detected; FAE—ferulic acid equivalents; QE—quercetin equivalents; CGAE—chlorogenic acid equivalents.

According to Figure 2a–c, where values of TPC, TFC and HCAs in the leaves of *A. schoenoprasum* are shown, it was established that there was a statistically significant difference in the content of the observed parameters for both growing seasons, except in the case of TPC and TFC in the treatments with 20 and 30 g of Se fertilizer per ha.

In the first growing season, statistically higher TPC and TFC were recorded in the treatment with 10 g of Se per ha and in the treatment with 20 g of Se per ha for HCAs compared to the control condition and other treatments. In the second growing season, the highest and statistically significantly different TPC was recorded with the treatment of 20 g Se per ha compared to the content measured in other treatments. In addition, the same trend was observed for HCA values as was previously reported [30]. Interestingly, the same trend was perceived in the case of TFC—the highest content was measured in the treatment with 20 g of Se fertilizer per ha; however it was not statistically significantly different compared to the content observed in the treatment with 10 g Se per ha.

## 3. Discussion

### 3.1. Elemental Profile and Bioaccumulation Abilities 

The importance of Se for plant nutrition is still controversial, while its importance to humans and animals is well-studied. Considering the fact that food is the major source of Se in human nutrition, plants as an indispensable part of the diet are an excellent tool for raising the level of Se in the human body [31]. According to Navarro-Alarcon and Cabrera-Vique [32], the absorption of selenium from food is about 80%. Plants from the *Allium* genus are recognized as ‘natural dietary Se supplements’ due to their excellent ability to accumulate Se [33]. About that fact, biofortification has been recognized as an agrotechnical measure that can enrich the chemical composition of these plants, which would potentially increase their already high presence in the diet. 

Plants take up Se mainly as inorganic ions, namely, selenate or selenite, which are the most common forms of Se in the soil. Due to similar chemical properties, selenate and sulphate are competitors in the processes of assimilation, translocation and metabolism [34]. Moreover, the uptake of selenate and sulphate through the roots is carried out by the same transporters, and Se is further metabolized by S pathways. Competition between S and Se, according to White et al. [35], is determined by several factors: plant species, Se/S ratio in the soil as well as Se/S ratio in plant tissues. The same author pointed out that there is often no correlation between Se and S content in plant tissues, even though the plants are grown in the same conditions [35]. However, different results were obtained in agricultural research. In the study by Mobini et al. [36] where onion was grown with the application of Se and S to the nutrient solution, it was determined that the addition of Se had no significant effect on the content of S in the bulbs, while the addition of S (1–3 mM) led to a decrease in Se content in the bulbs. On the other hand, a study by Kopsell and Randle [37] indicated that with the increase of Se concentration, the content of S in the bulbs of different onion cultivars decreased. Research of Tian et al. [38] showed that application of Na_2_SeO_3_ in nutrient solution (20–40 µM) led to a decrease in total S content in the shoot of broccoli, while the application of Na_2_SeO_4_ (20 µM) significantly enhanced the total S content. In the current research, the foliar application of Se fertilizer in the form of sodium selenate (Na_2_SeO_4_) contributed to a significant increase in the content of this element in the edible parts (leaves) of *A. odorum* and *A. schoenoprasum* in both seasons. The observed results are in agreement with the results reported from Estonia, where the foliar application of Se fertilizer on *A. sativum* led to an increase in the content of Se in the bulbs, with an increased dose of Se fertilizer [39]. Similar results were obtained in previously published studies conducted on onion, garlic and lettuce [37,40,41]. 

In general, in the first season, the application of Se fertilizer at a dose of 10 g per ha proved to be adequate for the majority of examined elements for both *Allium* species. In particular, this was observed for microelement content. In the second season, higher doses of Se fertilizer promoted better accumulation of the tested biogenic elements. More precisely, a dose of 30 g of Se fertilizer per ha was optimal for *A. odorum* and 20 g per ha for *A. schoenoprasum.* In research conducted on garlic it was shown that foliar treatment of Se fertilizer, especially higher doses (50 and 100 µg mL^−1^), led to a decrease in the content of macroelements—N, P, K, Ca [39]. The observed trend was presented in the current research for P and K. The same authors indicated that no negative correlation was found between the dose of Se and the content of macroelements in onion [42]. 

The bioaccumulation factor (BAF), as an indicator of plants’ ability to accumulate micro, macro and potentially toxic elements from the soil, indicates that the examined *Allium* species differ. Namely, both *Allium* species (*A. odorum* and *A. schoenoprasum*) accumulated S, K and P in the edible parts (leaves) in both seasons. These elements participate in many vital processes in mammals and plants. Sulfur (S) is an integral component of amino acids (methionine and cysteine). In plants, S is a constituent of enzymes important for photosynthesis, respiration and the synthesis of fatty acids; it affects seed quality; it is important for membrane transport; and it reduces stress caused by salt and toxic metals. Similarly, K contributes to photosynthesis, transpiration, growth and development, and it reduces the effects of drought stress. Furthermore, P affects processes related to yield and quality (bud formation, starch accumulation, fruit ripening) [15]. 

In this research it was observed that *A. odorum* had a higher affinity for accumulation of the mentioned macroelements compared to *A. schoenoprasum*. In addition, the effect of the growing season was not pronounced in *A. odorum*, whereas in *A. schoenoprasum,* during the second season, which was marked as less favorable for most agricultural crops [43], BAF values were lower compared to the values recorded in the first season. According to Liang et al. [44], this result can be explained as a consequence of drought stress. Based on the agrometeorological conditions described in Section 4.2., Season II was characterized by a significantly lower amount of rainfall (around 200 mm lower), confirming that it was indeed a season with less favorable conditions and possible exposure of plants to drought stress conditions. More specifically, in drought conditions, the uptake, transport and generally the concentration of macro, micro and trace elements in the leaves and stems are lower compared to normal growth conditions. In a study by Golubkina et al. [45], different capacities of perennial *Allium* species to accumulate biogenic elements are explained by differences in leaf surface and adhesion abilities. In a study by Zhang et al. [46], it was found that foliar-applied substances predominantly could be infiltrated by the cuticle and stomata and before being taken up by inner leaf tissue. Therefore, the nutrient pathway mainly depends on the leaf morphology and structure, number of stomata per leaf area and thickness of the cuticle of *Allium*. Stomata functioning is hugely impacted by many physiological parameters, such as water status, vapor-pressure deficit, photosynthetic activity and phytohormones, e.g., ABA, a well-known stress hormone that can induce stomatal closure under environmental stress induced by temperature, drought, etc. [47]. In a study by Zhang et al. [46], ABA was applied to induce stomatal closure on wheat leaves, and as a consequence, selenite absorption was greatly inhibited. Cuticles represent the second way of nutrient diffusion through the leaf. Water solution together with Se particles can diffuse in a passive way through the cuticles, especially with increased exogenous selenite concentrations, and the rate of selenite in inner leaf cells mainly depends on the penetration rate through the cuticle and stomata. Furthermore, according to BAF values (Table 3 and Table 5), both species did not accumulate potentially toxic elements in the edible parts, which makes them a safe source of biogenic elements for human nutrition. Results of our previously reported research indicated the presence of potentially toxic elements in garlic bulbs, but in allowed contents [48].

### 3.2. General Phytochemical Composition

*Allium* species are known as rich sources of phenolic compounds, which comprise an important class of secondary metabolites (SMs). The largest class of phenolics is the flavonoids, followed by phenolic acids and lignans [49]. They play crucial roles in the developmental and metabolic processes of the plant, participate in the interaction of plants with the environment and are responsible for the adaptation of plants in stress conditions [10,11]. In addition, they are closely associated with numerous biological actions important for human health—from antioxidant to anticancer. In the current study, a positive effect of foliar application of Se on SM content was determined. In general, for both *Allium* species, TPC, TFC and HCAs were higher in treatments with foliar application of Se (10, 20 and 30 g per ha) compared to control conditions. Our results are in agreement with the results published by Skrypnik et al. [50], in which it was found that the application of sodium selenate (Na_2_SeO_4_), either through a nutrient solution or foliarly, increases the content of SMs in *Hyssopus officinalis*. In the research of Astaneh et al. [51], it was observed that Se application through a nutrient solution significantly promoted the accumulation of TPC in garlic leaves, which were grown hydroponically, in a greenhouse, under NaCl stress. However, treatment with Na_2_SeO_4_ did not have a statistically significant effect on increasing the content of phenolics and flavonoids in shallots according to research done by Golubkina et al. [52]. Furthermore, research conducted under open field conditions showed that the application of selenium significantly reduced the content of flavonoids in garlic compared to control plants [53]. These contradictory results can be explained by different growing conditions during the extent of the experiments, as well as different concentrations and methods of applying Se fertilizer. In addition, they confirm the need for further experiments. 

In addition, in the sequence of TPC, TFC and HCAs, a significant influence of the growing season was determined. Precisely, during the second season when the plants were exposed to drought stress, a higher content of the mentioned parameters was recorded. These results were in accordance with many review studies that described a positive correlation between SM content and the impact of abiotic and biotic stress. In fact, the synthesis of these functional molecules allows the plant to counteract the negative impacts caused by stressful conditions [11,12,13,54]. 

## 4. Materials and Methods

### 4.1. Experimental Field and Plant Material 

A two-year experiment was conducted during October 2019/July 2020 (Season I) and October 2020/July 2021 (Season II), in open field conditions, in Belgrade (44°54′31.3″ N 20°25′42.1″ E), Serbia. The experiment was arranged in three replicates to test the effects of foliar application of Se fertilizer on selected *Allium* species. 

Plant seedlings of *A. odorum* and *A. schoenoprasum* were provided by the Institute of Field and Vegetable Crops from Novi Sad. Planting was carried out in October with a plant density of 30 plants per m^2^.

**Figure 3:**
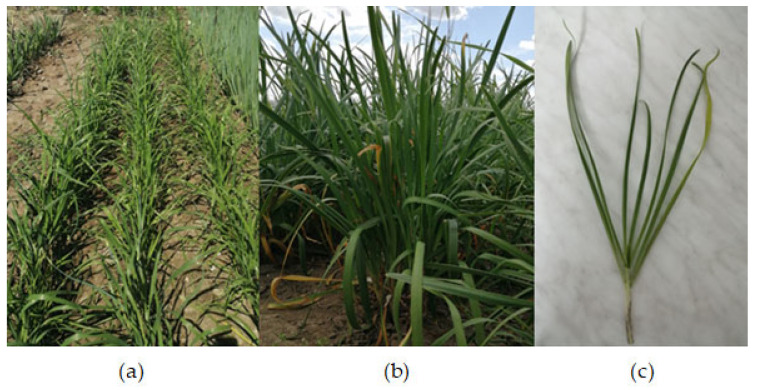
Appearance of *A. odorum* (**a**,**b**); edible parts (leaves) of *A. odorum* (**c**).

**Figure 4:**
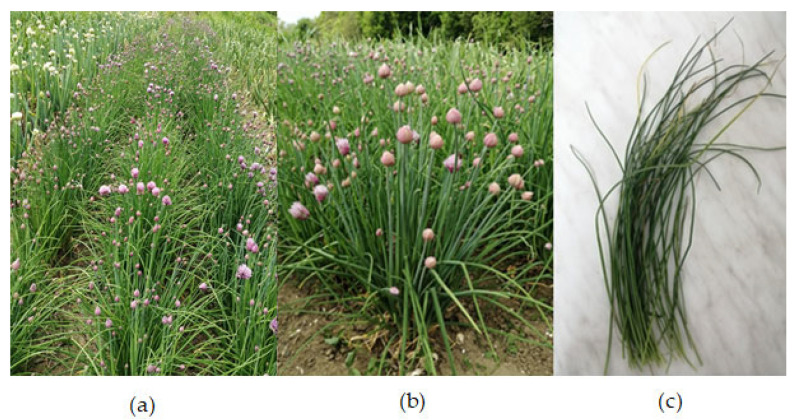
Appearance of *A. schoenoprasum* (**a**,**b**); edible parts (leaves) of *A. schoenoprasum* (**c**).

During the experiment, the following plant care measures were performed: mechanical weed control, protection from diseases and pests and watering as needed. The foliar application of Se fertilizer as a water solution of sodium-selenate (Na_2_SeO_4_) was conducted in the phase of intensive growth of *Allium* species (April in both seasons, Figure 3 and Figure 4a,b). Na_2_SeO_4_ was applied in three doses—10, 20 and 30 g per ha. In the control condition, the plants were not treated with Na_2_SeO_4_.

Edible parts (leaves) (Figure 3 and Figure 4c) were collected using a completely random method in the flowering phase for both tested species. Preparation of plant material included cleaning the material of soil, washing, chopping and freezing at 18 °C. Samples were kept frozen until preparation for microwave digestion and further analysis.

### 4.2. Growth Conditions

Meteorological parameters for both production seasons were measured by a meteorological station in Belgrade, and the data were taken from the Republic Hydrometeorological Service of Serbia [43].

In Season I (October 2019/July 2020), the average daily temperature (ADT) for the Belgrade region was 15 °C, taking into account that for the period October–March, the temperature deviated by 3.4 °C from the ADT, while for the period April–September, the deviation was 1.2 °C. The total rainfall for Season I was 635 mm. The total insolation during Season I was 2300 h for the Belgrade region. In general, the vegetation period in 2020 (April–September) was warmer and more frequently humid compared to average conditions.

In Season II (October 2020/July 2021), the ADT was 14 °C, with deviation of 1.7 °C for October–March and 1.3 °C for April–September. The total rainfall was 462 mm, and the total insolation was 2300 h. In summary, the vegetation period in 2021 (April–September) was warmer and drier compared to average conditions.

Briefly, the production year 2019/2020, from the point of view of agrometeorological conditions, was appropriate for most agricultural crops, while the production year 2020/2021 was not favorable for many crops.

### 4.3. Agrochemical Soil Analysis

The agrochemical soil analysis was performed before setting up the experiment. The soil was sampled at a depth of 0–30 cm, and agrochemical analysis was performed by means of common methods, in accordance with ISO standards. The soil of the experimental field was chernozem semigley [55] with the agrochemical properties listed in Table 6.

According to the mentioned properties, the tested soil was classified as neutral to slightly alkaline [56], medium humic [57] and medium carbonate. The C:N ratio (9.8:1) indicated that the processes of C oxidation and N immobilization were in equilibrium [58]. According to N content (including ammonium and nitrate), the tested soil was sufficiently supplied with it, while the content of P and K was higher than the recommended thresholds [59].

### 4.4. Determination of the Content of Biogenic Elements in the Soil and Plant Material by Inductively Coupled Plasma–Optical Emission Spectroscopy (ICP-OES)

The elemental profile of the examined *Allium* species was determined by application of the ICP-OES technique with previously digested samples. The digestion of the samples was performed on an Advanced Microwave Digestion System (Ethos 1, Milestone, Italy) using an HPR-1000/10S high pressure segmented rotor. The pressure-resistant PTFE vessels (volume 100 mL), which were equipped with QS-50 Quartz inserts, were used. About 2 g of the sample was precisely weighed with an accuracy of ±0.1 mg, placed in the quartz insert and mixed with 5 mL of HNO*_3_* (65 wt.%, Suprapur^®^) and 0.5 mL H*_2_*O*_2_* (30 wt.%, Suprapur^®^) (both of Merck KGaA, Darmstadt, Germany). The temperature was gradually raised with microwave power (0–1000 W): linearity from 25 to 180 ºC in the first 15 min, maintained at 180 °C for the next 20 min and then decreased rapidly to room temperature. After cooling and without filtration, the solution was diluted to a fixed volume (25 mL) in the volumetric flask with ultrapure water. Ultrapure water with a conductivity of 0.05 µS/cm was prepared using a Barnstead™ GenPure™ Pro (Thermo Scientific, Karlsruhe, Germany).

The contents of macro and microelements in solution samples were determined by inductively coupled plasma–optical emission spectrometry (iCAP 6500 Duo ICP, Thermo Fisher Scientific, Cambridge, UK). The external calibration solutions were made from three certified plasma standard solutions: Multi-Element Plasma Standard Solution 4, Specpure^®^, 1000 µg/mL (Alfa Aesar GmbH & Co. KG, Kandel, Germany) and SS-Low Level Elements ICV Stock and ILM 05.2 ICS Stock 1 (both of VHG Labs, Inc.—Part of LGC Standards, Manchester, NH 03103, USA). Quality control was carried out using blank samples, matrix-matched calibration solutions and triplicate analyses of each sample. The reliability of measurements was approved by a relative standard deviation (RSD) < 1%. The analytical process quality control was performed by using two certified reference materials (CRMs) of fish protein for trace metals DORM 4 (NRCC, National Research Council Canada, Ottawa, ON, Canada) and EPA Method 200.7 LPC Solution (ULTRA Scientific, North Kingstown, RI, USA). Recovery of measured concentrations with certified values was 97–103. Concentrations of elements in the sample were expressed as µg g^−1^ of fresh weight.

### 4.5. Determination of Bioaccumulation Factor (BAF)

BAF indicates the ability of a plant to accumulate elements present in the soil in overground or/and underground organs. It represents the ratio of a certain element in the plant organ and in the soil. It is calculated according to the following equation:

$$\mathrm{BAF}=\frac{Cp}{Cs}$$

where Cp —concentration of a certain element in the tissues of the plant overground/underground organs (mg kg^−1^), Cs —concentration of a certain element in the soil (mg kg^−1^) [60].

BAF values above 1 indicate the efficiency of the plant to accumulate a certain element [61]. According to another categorization, BAF < 1 indicates excluder, 1–10 accumulator and >10 hyperaccumulator plants [62].

### 4.6. Determination of the Content of Total Free Phenolics (TPC), Total Free Flavonoids (TFC) and Free Hydroxycinnamic Acid Derivatives (HCAs)

Determination of TPC, TFC and HCAs was done by spectrophotometric methods (apparatus model: UV-1800, Shimadzu USA Manufacturing Inc., UR, USA), as previously described by Kilibarda et al. [63]. The results were expressed as mg of ferulic acid equivalents (FAE), mg of quercetin equivalents (QE) and mg of chlorogenic acid equivalents (CGAE) per g of fresh weight (FW), respectively.

### 4.7. Data Analysis

The data were analyzed by a one-way analysis of variance (ANOVA) using R software, version 3.6.0 [64]. Evaluation of statistical significance between different levels of Se fertilizers was performed using Tukey’s HSD test. Statistical significance was considered at *p* < 0.05. All tests were performed in triplicate, and the results were expressed as mean ± standard deviation (SD).

## 5. Conclusions

The study of Se-biofortification on rare *Allium* species (*A. odorum* and *A. schoenoprasum*) in Serbia, under open field conditions, during two growing seasons included determination of the elemental profile and general phytochemical composition of plants. For the content of biogenic elements (macro and microelements), the application of selenium as foliar fertilizer seemed to be justified, since it enhanced the determined content for most of the elements but with the strong influence of open field growing conditions. BAF values indicated the ability of both species to accumulate S, K and P in the leaves in both seasons. According to general phytochemical composition (TPC, TFC and HCAs), the effect of Se depended mostly on the growing season; i.e., the content of the mentioned parameters was statistically significantly higher in the second season compared to the first one. The observed trend was mostly influenced by drought stress in the second season. Based on the obtained data, further research should be focused on extended research with different Se doses in order to find the most favorable to ensure the optimal content of biogenic elements and SMs important for human health, and which would also be economically profitable. Growing the examined alliums in controlled conditions and with different methods of selenium enrichment are the next goals for research.

## Figures and Tables

**Table 1:** The contents of macro and microelements in the soil in the experimental field.

Element	Measured Value in the Soil (mg kg^−1^)	Recommended Range in the Soil (mg kg^−1^) with References
Al	17,200.00	10,000.00–30,000.00 [23]
As	8.62	29.00 [24]
B	14.04	10.00–100.00 [25]
Ba	141.63	160.00 [24]
Ca	24,950.50	1700.00–104,000.00 [26]
Cd	0.16	0.80 [24]
Co	9.90	4.5–12.00 [25]
Cr	21.39	100.00 [24]
Cu	48.52	8.00–80.00 [25]
Fe	29,300.00	1000.00–49,000.00 [25]
K	2739.50	300.00–32,000.00 [27]
Li	25.59	7.00–200.00 [28]
Mg	10,733.04	2000.00–12,600.00 [26]
Mn	658.40	40.00–900.00 [29]
Na	365.35	1340.00–15,800.00 [26]
Ni	37.07	5.00–90.00 [25]
P	1052.48	500.00–800.00 [27]
Pb	20.20	85.00 [24]
S	578.22	100.00–1000.00 [27]
Se	<1.00	0.70 [24]
Sr	48.51	40.00–250.00 [25]
Zn	121.80	10.00–300.00 [25]

**Table 2:** The contents (µg g^−1^ of fresh weight ± SD) of biogenic elements observed in the leaves of *A. odorum* for both seasons.

	Season I				Season II				
Element	Doses of Se Fertilizer				Element	Doses of Se Fertilizer			
	Control	10 g ha^−1^	20 g ha^−1^	30 g ha^−1^		Control	10 g ha^−1^	20 g ha^−1^	30 g ha^−1^
**Al**	5.60 ± 0.26 c *	8.67 ± 0.27 a	6.41 ± 0.23 b	5.31 ± 0.20 c	**Al**	2.04 ± 0.04 b	2.20 ± 0.05 b	2.42 ± 0.13 a	2.81 ± 0.07 a
**As**	n.d. **	n.d.	0.01 ± 0.00	n.d.	**As**	n.d.	n.d.	n.d.	n.d.
**B**	3.24 ± 0.16 b	3.81 ± 0.14 a	2.03 ± 0.02 d	2.31 ± 0.10 c	**B**	1.11 ± 0.06 b	1.19 ± 0.05 b	0.98 ± 0.01 c	1.37 ± 0.06 a
**Ba**	1.53 ± 0.06 b	1.81 ± 0.08 a	1.64 ± 0.07 ab	1.75 ± 0.07 a	**Ba**	2.16 ± 0.06 a	1.90 ± 0.06 b	2.08 ± 0.04 a	1.67 ± 0.09 c
**Ca**	873.91 ± 34.15 c	1414.51 ± 59.13 a	1058.07 ± 47.32 b	1031.02 ± 46.25 b	**Ca**	699.43 ± 23.22 c	723.87 ± 27.18 c	851.29 ± 12.02 b	927.03 ± 14.50 a
**Cd**	0.02 ± 0.00 a	0.01 ± 0.00 b	0.02 ± 0.00 a	0.02 ± 0.00 a	**Cd**	0.01 ± 0.00 a	0.01 ± 0.00 a	0.01 ± 0.00 a	0.01 ± 0.00 a
**Co**	n.d.	n.d.	n.d.	n.d.	**Co**	n.d.	n.d.	n.d.	n.d.
**Cr**	0.06 ± 0.00 b	0.08 ± 0.00 a	0.06 ± 0.00 b	0.08 ± 0.00 a	**Cr**	0.04 ± 0.00 a	0.04 ± 0.00 a	0.03 ± 0.00 b	0.04 ± 0.00 a
**Cu**	0.70 ± 0.02 a	0.67 ± 0.02 a	0.53 ± 0.03 b	0.55 ± 0.02 b	**Cu**	0.54 ± 0.01 c	0.54 ± 0.01 c	0.56 ± 0.01 b	0.58 ± 0.01 a
**Fe**	7.77 ± 0.34 c	11.37 ± 0.38 a	8.66 ± 0.26 b	8.06 ± 0.05 bc	**Fe**	4.95 ± 0.10 d	5.19 ± 0.04 c	6.06 ± 0.02 b	6.49 ± 0.07 a
**K**	3142.86 ± 29.21 b	2955.25 ± 32.96 c	3456.52 ± 21.13 a	3038.58 ± 22.77 c	**K**	4884.29 ± 25.04 a	4641.24 ± 34.21 c	4774.29 ± 24.79 b	4524.71 ± 21.34 d
**Li**	0.04 ± 0.00 c	0.07 ± 0.00 a	0.05 ± 0.00 b	0.05 ± 0.00 b	**Li**	0.03 ± 0.00 c	0.04 ± 0.00 b	0.04 ± 0.00 b	0.05 ± 0.00 a
**Mg**	354.35 ± 14.95 bc	319.75 ± 14.25 c	354.97 ± 17.52 b	404.17 ± 17.34 a	**Mg**	268.14 ± 10.11 c	292.66 ± 12.70 c	346.86 ± 10.96 a	312.94 ± 9.96 b
**Mn**	1.94 ± 0.05 a	1.64 ± 0.08 b	1.63 ± 0.07 b	1.63 ± 0.06 b	**Mn**	1.03 ± 0.06 b	1.06 ± 0.03 b	1.28 ± 0.03 a	1.19 ± 0.03 a
**Na**	18.77 ± 0.54 b	19.58 ± 0.30 b	14.11 ± 0.37 c	21.42 ± 0.42 a	**Na**	n.d.	1.20 ± 0.03 c	3.26 ± 0.03 a	1.30 ± 0.03 b
**Ni**	0.07 ± 0.00 b	0.06 ± 0.00 c	0.08 ± 0.00 a	0.04 ± 0.00 d	**Ni**	0.06 ± 0.00 b	0.02 ± 0.00 c	0.07 ± 0.00 a	0.02 ± 0.00 c
**P**	642.24 ± 28.47 c	582.87 ± 24.63 c	893.48 ± 34.27 a	749.69 ± 34.53 b	**P**	698.43 ± 10.23 c	727.97 ± 10.63 b	682.71 ± 10.76 c	1027.76 ± 11.70 a
**Pb**	0.03 ± 0.00 c	0.07 ± 0.00 a	0.02 ± 0.00 d	0.05 ± 0.00 b	**Pb**	n.d.	n.d.	n.d.	n.d.
**S**	868.17 ± 39.53 a	677.16 ± 30.95 bc	638.04 ± 28.31 c	732.87 ± 23.26 b	**S**	721.00 ± 26.61 ab	721.75 ± 33.44 ab	764.57 ± 18.44 a	669.91 ± 21.23 b
**Se**	0.04 ± 0.00 d	0.13 ± 0.01 c	0.50 ± 0.02 b	0.57 ± 0.03 a	**Se**	0.18 ± 0.01 d	0.47 ± 0.02 c	0.82 ± 0.02 b	2.52 ± 0.05 a
**Sr**	2.59 ± 0.12 b	3.15 ± 0.05 a	2.76 ± 0.10 b	2.88 ± 0.09 b	**Sr**	2.63 ± 0.04 b	2.72 ± 0.12 b	2.92 ± 0.05 a	2.56 ± 0.12 b
**Zn**	2.61 ± 0.13 ab	2.11 ± 0.09 c	2.85 ± 0.12 a	2.57 ± 0.12 b	**Zn**	2.17 ± 0.11 b	2.64 ± 0.12 a	2.20 ± 0.05 b	2.82 ± 0.09 a

* Different letters in the same row in each season indicate significant differences according to Tukey’s HSD test (*p* ≤ 0.05); ** n.d.—not detected.

**Table 3:** Bioaccumulation factor (BAF) values for biogenic elements in the leaves of *A. odorum* for both seasons.

Season I					Season II				
Element	Doses of Se Fertilizer				Element	Doses of Se Fertilizer			
	Control	10 g ha^−1^	20 g ha^−1^	30 g ha^−1^		Control	10 g ha^−1^	20 g ha^−1^	30 g ha^−1^
**Al**	3.26 × 10^−4^	0.0005	0.0004	0.0003	**Al**	1.19 × 10^−4^	0.0001	0.0001	0.0002
**As**	n.d. *	n.d.	0.00	n.d.	**As**	n.d.	n.d.	n.d.	n.d.
**B**	0.2310	0.2717	0.1447	0.1648	**B**	0.0793	0.0847	0.0698	0.0977
**Ba**	0.0108	0.0128	0.0116	0.0123	**Ba**	0.0153	0.0134	0.0147	0.0118
**Ca**	0.3502	0.5668	0.4240	0.4132	**Ca**	0.2803	0.2901	0.3411	0.3715
**Cd**	0.110	0.088	0.104	0.116	**Cd**	0.0795	0.0620	0.0689	0.0822
**Co**	n.d.	n.d.	n.d.	n.d.	**Co**	n.d.	n.d.	n.d.	n.d.
**Cr**	0.0029	0.0037	0.0027	0.0037	**Cr**	0.0017	0.0018	0.0013	0.0019
**Cu**	0.0145	0.0139	0.0110	0.0114	**Cu**	0.0110	0.0111	0.0116	0.0119
**Fe**	0.0003	0.0004	0.0003	0.0003	**Fe**	0.0002	0.0002	0.0002	0.0002
**K**	1.1472	1.0788	1.2617	1.1092	**K**	1.7829	1.6942	1.7428	1.6517
**Li**	0.0016	0.0025	0.0018	0.0018	**Li**	0.0014	0.0014	0.0017	0.0018
**Mg**	0.0330	0.0298	0.0331	0.0377	**Mg**	0.0250	0.0273	0.0323	0.0292
**Mn**	0.0029	0.0025	0.0025	0.0025	**Mn**	0.0016	0.0016	0.0019	0.0018
**Na**	0.0514	0.0536	0.0386	0.0586	**Na**	n.d.	0.0033	0.0089	0.0036
**Ni**	0.0020	0.0017	0.0021	0.0010	**Ni**	0.0015	0.0005	0.0019	0.0004
**P**	0.6102	0.5538	0.8489	0.7123	**P**	0.6636	0.6917	0.6487	0.9765
**Pb**	0.0013	0.0036	0.0011	0.0026	**Pb**	n.d.	n.d.	n.d.	n.d.
**S**	1.5015	1.1711	1.1035	1.2675	**S**	1.2469	1.2482	1.3223	1.1586
**Se**	n.d.	n.d.	n.d.	n.d.	**Se**	n.d.	n.d.	n.d.	n.d.
**Sr**	0.0534	0.0649	0.0569	0.0593	**Sr**	0.0542	0.0561	0.0602	0.0527
**Zn**	0.0215	0.0173	0.0234	0.0211	**Zn**	0.0178	0.0217	0.0181	0.0231

* n.d.—not determined.

**Table 4:** The contents (µg g^−1^ of fresh weight ± SD) of biogenic elements observed in the leaves of *A. schoenoprasum* for both seasons.

	Season I				Season II				
Element	Doses of Se Fertilizer				Element	Doses of Se Fertilizer			
	Control	10 g ha^−1^	20 g ha^−1^	30 g ha^−1^		Control	10 g ha^−1^	20 g ha^−1^	30 g ha^−1^
**Al**	14.58 ± 0.46 b*	13.23 ± 0.24 c	17.02 ± 0.52 a	9.95 ± 0.23 d	**Al**	9.84 ± 0.36 b	5.98 ± 0.21 c	17.08 ± 0.05 a	4.68 ± 0.23 d
**As**	n.d. **	n.d.	n.d.	n.d.	**As**	n.d.	n.d.	n.d.	n.d.
**B**	2.87 ± 0.11 b	3.46 ± 0.16 a	2.76 ± 0.11 b	2.72 ± 0.10 b	**B**	0.86 ± 0.02 a	0.62 ± 0.00 c	0.77 ± 0.03 b	0.59 ± 0.01 c
**Ba**	1.50 ± 0.07 a	1.43 ± 0.05 a	1.41 ± 0.07 a	1.58 ± 0.06 a	**Ba**	1.09 ± 0.00 b	0.92 ± 0.03 c	1.14 ± 0.02 a	1.06 ± 0.03 b
**Ca**	1800.93 ± 72.85 ab	1900.62 ± 91.45 a	1677.02 ± 50.23 b	1858.70 ± 77.08 ab	**Ca**	1681.69 ± 48.32 a	1473.99 ± 46.62 b	1792.13 ± 79.46 a	1528.27 ± 64.86 b
**Cd**	0.03 ± 0.00 a	0.03 ± 0.00 a	0.03 ± 0.00 a	0.03 ± 0.00 a	**Cd**	0.02 ± 0.00 a	0.02 ± 0.00 a	0.02 ± 0.00 a	0.02 ± 0.00 a
**Co**	n.d.	n.d.	n.d.	n.d.	**Co**	n.d.	n.d.	n.d.	n.d.
**Cr**	0.09 ± 0.00 a	0.09 ± 0.00 a	0.09 ± 0.00 aa	0.05 ± 0.00 b	**Cr**	0.09 ± 0.00 b	0.05 ± 0.00 c	0.11 ± 0.00 a	0.09 ± 0.00 b
**Cu**	0.65 ± 0.03 b	0.67 ± 0.03 b	0.76 ± 0.03 a	0.55 ± 0.03 c	**Cu**	0.35 ± 0.01 a	0.30 ± 0.02 c	0.31 ± 0.02 bc	0.33 ± 0.00 ab
**Fe**	17.82 ± 0.63 b	17.47 ± 0.40 b	19.64 ± 0.73 a	12.38 ± 0.33 c	**Fe**	11.13 ± 0.00 b	8.36 ± 0.21 c	20.77 ± 0.06 a	8.37 ± 0.02 c
**K**	2560.19 ± 116.37 a	2414.60 ± 108.79 a	2586.96 ± 102.79 a	2450.31 ± 96.38 a	**K**	2271.80 ± 21.78 a	2117.05 ± 16.16 bc	2129.21 ± 7.84 b	2069.94 ± 19.16 c
**Li**	0.09 ± 0.00 a	0.09 ± 0.00 aa	0.08 ± 0.00 b	0.08 ± 0.00 b	**Li**	0.09 ± 0.00 b	0.08 ± 0.00 b	0.11 ± 0.00 a	0.07 ± 0.00 c
**Mg**	231.02 ± 6.97 ab	248.45 ± 10.55 a	224.84 ± 10.19 ab	214.29 ± 9.83 b	**Mg**	238.08 ± 3.74 a	184.97 ± 4.15 c	198.60 ± 4.98 b	193.75 ± 3.05 bc
**Mn**	1.87 ± 0.07 b	2.35 ± 0.07 a	2.18 ± 0.11 a	1.85 ± 0.09 b	**Mn**	1.19 ± 0.05 b	1.11 ± 0.02 c	1.42 ± 0.03 a	1.19 ± 0.04 b
**Na**	14.71 ± 0.52 a	12.25 ± 0.16 b	15.14 ± 0.46 a	12.59 ± 0.16 b	**Na**	5.77 ± 0.05 a	4.02 ± 0.27 b	4.10 ± 0.05 b	4.37 ± 0.04 b
**Ni**	0.13 ± 0.01 b	0.24 ± 0.01 a	0.14 ± 0.01 b	0.15 ± 0.01 b	**Ni**	0.25 ± 0.01 a	0.02 ± 0.00 d	0.16 ± 0.01 b	0.04 ± 0.00 c
**P**	628.70 ± 23.94 b	746.58 ± 22.05 a	661.02 ± 21.22 b	657.61 ± 22.73 b	**P**	366.72 ± 9.73 a	327.60 ± 2.86 b	296.63 ± 8.96 c	367.86 ± 5.36 a
**Pb**	0.05 ± 0.00 b	0.06 ± 0.00 a	0.05 ± 0.00 b	0.01 ± 0.00 c	**Pb**	n.d.	n.d.	n.d.	n.d.
**S**	737.96 ± 26.09 b	727.80 ± 20.00 b	790.37 ± 17.06 a	787.58 ± 19.69 a	**S**	428.63 ± 8.13 b	436.71 ± 4.82 b	466.57 ± 5.34 a	451.04 ± 13.64 a
**Se**	0.03 ± 0.00 d	1.12 ± 0.05 c	2.70 ± 0.12 b	4.10 ± 0.13 a	**Se**	0.51 ± 0.01 d	0.73 ± 0.03 c	0.89 ± 0.01 b	1.53 ± 0.07 a
**Sr**	4.76 ± 0.22 a	4.59 ± 0.16 a	4.47 ± 0.22 a	4.83 ± 0.15 a	**Sr**	4.20 ± 0.08 b	4.08 ± 0.09 b	4.75 ± 0.21 a	4.43 ± 0.11 a
**Zn**	2.85 ± 0.11 b	3.12 ± 0.15 a	2.86 ± 0.09 b	2.85 ± 0.13 b	**Zn**	1.22 ± 0.06 b	0.98 ± 0.03 c	1.22 ± 0.08 b	1.27 ± 0.04 a

* Different letters in the same row in each season indicate significant differences, according to Tukey’s HSD test (*p* ≤ 0.05); ** n.d.—not detected.

**Table 5:** Bioaccumulation factor (BAF) values for biogenic elements in the leaves of *A. schoenoprasum* for both seasons.

Season I					Season II				
Element	Doses of Se Fertilizer				Element	Doses of Se Fertilizer			
	Control	10 g ha^−1^	20 g ha^−1^	30 g ha^−1^		Control	10 g ha^−1^	20 g ha^−1^	30 g ha^−1^
**Al**	8.47 × 10^−4^	0.0008	0.0010	0.0006	**Al**	5.72 × 10^−4^	0.0003	0.0010	0.0003
**As**	n.d. *	n.d.	n.d.	n.d.	**As**	n.d.	n.d.	n.d.	n.d.
**B**	0.2044	0.2465	0.1969	0.1939	**B**	0.0609	0.0444	0.0548	0.0423
**Ba**	0.0106	0.0101	0.0100	0.0111	**Ba**	0.0077	0.0065	0.0081	0.0075
**Ca**	0.0722	0.0762	0.0672	0.0745	**Ca**	0.0674	0.0591	0.0718	0.0613
**Cd**	0.1867	0.2008	0.1978	0.1685	**Cd**	0.1224	0.0940	0.1162	0.1210
**Co**	n.d.	n.d.	n.d.	n.d.	**Co**	n.d.	n.d.	n.d.	n.d.
**Cr**	0.0041	0.0040	0.0041	0.0024	**Cr**	0.0040	0.0024	0.0053	0.0041
**Cu**	0.0134	0.0148	0.0157	0.0114	**Cu**	0.0072	0.0061	0.0063	0.0068
**Fe**	0.0006	0.0006	0.0007	0.0004	**Fe**	0.0004	0.0003	0.0007	0.0003
**K**	0.9345	0.8814	0.9443	0.8944	**K**	0.8293	0.7728	0.7772	0.7556
**Li**	0.0034	0.0037	0.0033	0.0032	**Li**	0.0034	0.0030	0.0042	0.0029
**Mg**	0.0215	0.0231	0.0209	0.0200	**Mg**	0.0222	0.0172	0.0185	0.0181
**Mn**	0.0028	0.0036	0.0033	0.0028	**Mn**	0.0018	0.0017	0.0022	0.0018
**Na**	0.0403	0.0335	0.0414	0.0345	**Na**	0.0158	0.0110	0.0112	0.0120
**Ni**	0.0036	0.0065	0.0037	0.0040	**Ni**	0.0067	0.0005	0.0042	0.0011
**P**	0.5974	0.7094	0.6281	0.6248	**P**	0.3484	0.3113	0.2818	0.3495
**Pb**	0.0022	0.0028	0.0025	0.0004	**Pb**	n.d.	n.d.	n.d.	n.d.
**S**	1.2763	1.2587	1.3669	1.3621	**S**	0.7413	0.7553	0.8069	0.7801
**Se**	n.d.	n.d.	n.d.	n.d.	**Se**	n.d.	n.d.	n.d.	n.d.
**Sr**	0.0981	0.0945	0.0922	0.0996	**Sr**	0.0865	0.0841	0.0980	0.0914
**Zn**	0.0234	0.0256	0.0235	0.0234	**Zn**	0.0100	0.0081	0.0100	0.0105

* n.d.—not determined.

**Table 6:** Agrochemical properties of the soil sample from the experimental field.

**Properties of Soil Sample**	**pH_H2O_**	**pH_KCl_**	**Total Humus (%)**	**CaCO_3_** **(%)**	**NO_3-_–N** **(mg kg^−1^)**	**NH_4+_–N** **(mg kg^−1^)**	**P_2_O_5_** **(mg 100 g^−1^)**	**K_2_O** **(mg 100 g^−1^)**	**C:N**
	7.81	7.20	3.12	3.2	31.5	7.0	208.0	46.0	9.8:1

## Data Availability

Not applicable.

## References

[1] Li Q.Q., Zhou S.D., He X.J., Yu Y., Zhang Y.C., Wei X.Q. (2010). Phylogeny and biogeography of *Allium* (Amaryllidaceae: *Allieae*) based on nuclear ribosomal internal transcribed spacer and chloroplast rps16 sequences, focusing on the inclusion of species endemic to China. Ann. Bot..

[2] Marrelli M., Amodeo V., Statti G., Conforti F. (2018). Biological Properties and Bioactive Components of *Allium cepa* L.: Focus on Potential Benefits in the Treatment of Obesity and Related Comorbidities. Molecules.

[3] Najjaa H., Sami F., Ammar E., Neffati M., Valdez B. (2012). Allium Species, Ancient Health Food for the Future?. Scientific, Health and Social Aspects of the Food Industry.

[4] Upadhyay R.K. (2017). Nutritional and Therapeutic Potential of *Allium* Vegetables. J. Nutr. Ther..

[5] Hedges L., Lister C. (2007). The nutritional attributes of Allium species.

[6] Wang Y., Raghavan S., Ho C.T., Brückner B., Wyllie S.G. (2008). 11—Process flavors of *Allium* vegetables. Fruit and Vegetable Flavour.

[7] Kamenetsky R., Rabinowitch H.D., Thomas B., Murray B.G., Murphy D.J. (2017). Physiology of Domesticated Alliums: Onions, Garlic, Leek, and Minor Crops. Encyclopedia of Applied Plant Sciences.

[8] Bastaki S.M.A., Ojha S., Kalasz H., Adeghate E. (2021). Chemical constituents and medicinal properties of *Allium* species. Mol. Cell. Biochem..

[9] Fredotović Ž., Puizina J. (2019). Edible *Allium* species: Chemical composition, biological activity and health effects. Ital. J. Food Sci..

[10] Yang L., Wen K.-S., Ruan X., Zhao Y.-X., Wei F., Wang Q. (2018). Response of Plant Secondary Metabolites to Environmental Factors. Molecules.

[11] Sharma A., Shahzad B., Rehman A., Bhardwaj R., Landi M., Zheng B. (2019). Response of Phenylpropanoid Pathway and the Role of Polyphenols in Plants under Abiotic Stress. Molecules.

[12] Šamec D., Karalija E., Šola I., Vujčić Bok V., Salopek-Sondi B. (2021). The Role of Polyphenols in Abiotic Stress Response: The Influence of Molecular Structure. Plants.

[13] Toscano S., Trivellini A., Cocetta G., Bulgari R., Francini A., Romano D., Ferrante A. (2019). Effect of Preharvest Abiotic Stresses on the Accumulation of Bioactive Compounds in Horticultural Produce. Front. Plant Sci..

[14] Imelouane B., Tahri M., Elbastrioui M., Aouinti F., Elbachiri A. (2011). Mineral Contents of Some Medicinal and Aromatic Plants Growing in Eastern Morocco. J. Mater. Environ. Sci..

[15] Nieder R., Benbi D.K., Reichl F.X., Nieder R., Benbi D.K., Reichl F.X. (2018). Macro- and Secondary Elements and Their Role in Human Health. Soil Components and Human Health.

[16] Nieder R., Benbi D.K., Reichl F.X., Nieder R., Benbi D.K., Reichl F.X. (2018). Microelements and Their Role in Human Health. Soil Components and Human Health.

[17] Petropoulos S.A.-O., Fernandes Â., Ntatsi G., Petrotos K., Barros L., Ferreira I.A.-O. (2018). Nutritional Value, Chemical Characterization and Bulb Morphology of Greek Garlic Landraces. Molecules.

[18] Qaim M., Stein A.J., Meenakshi J.V. (2007). Economics of biofortification. J. Agric. Econ..

[19] Dhaliwal S.S., Sharma V., Shukla A.K., Verma V., Kaur M., Shivay Y.S., Nisar S., Gaber A., Brestic M., Barek V. (2022). Biofortification-A Frontier Novel Approach to Enrich Micronutrients in Field Crops to Encounter the Nutritional Security. Molecules.

[20] Shreenath A.P., Ameer M.A., Dooley J. (2022). Selenium Deficiency.

[21] Stefirta A., Botnari V., Brânza L., Bulhac I., Chilinciuc L. (2016). Agronomical biofortification of garlic plant (*Allium sativum* L.) in aspect of increasing selenium content and antioxidant properties. Adv. Med. Plant Res..

[22] Gupta M., Gupta S. (2017). An Overview of Selenium Uptake, Metabolism, and Toxicity in Plants. Front. Plant Sci..

[23] U.S. Environmental Protection Agency (2003). Appendix 5: US EPA Ecological Soil Screening Level for Aluminum.

[24] U.S. Environmental Protection Agency 2022. https://www.epa.gov/agriculture/agriculture-and-soils.

[25] Kabata-Pendias A., Mukherjee A.B. (2007). Trace Elements from Soil to Human.

[26] Jakovljevic M., Kostic N., Mladenovic S. (2003). The availability of base elements (Ca, Mg, Na, K) in some important soil types in Serbia. Zb. Matice Srp. Prir. Nauke.

[27] Mengel K., Kirkby E.A. (2004). Principles of plant nutrition. Ann. Bot..

[28] Bernard A., Nordberg G.F., Fowler B.A., Nordberg M. (2015). Chapter 44—Lithium. Handbook on the Toxicology of Metals.

[29] Lucchini R.G., Aschner M., Yangho K., Šarić M., Nordberg G.F., Fowler B.A., Nordberg M. (2015). Chapter 45—Manganese. Handbook on the Toxicology of Metals.

[30] Vuković S., Moravčević Đ., Gvozdanović-Varga J., Pećinar I., Vujošević A., Kilibarda S., Milinčić D.D., Gordanić S., Pavlović D., Kostić Ž.A. (2021). Hydroxycinnamic acid derivatives: Potential antioxidants in rare grown *Allium* species from Serbia. Book of Abstracts, Proceedings of the XII International Scientific Agriculture Symposium “AGROSYM 2021”, Jahorina, Bosnia and Hercegovina, 7–10 October 2021.

[31] Lavu R.V.S., Du Laing G., Van De Wiele T., Pratti V.L., Willekens K., Vandecasteele B., Tack F. (2012). Fertilizing Soil with Selenium Fertilizers: Impact on Concentration, Speciation, and Bioaccessibility of Selenium in Leek (*Allium ampeloprasum*). Agric. Food Chem..

[32] Navarro-Alarcon M., Cabrera-Vique C. (2008). Selenium in food and the human body: A review. Sci. Total Environ..

[33] Dumont E., Vanhaecke F., Cornelis R. (2006). Selenium speciation from food source to metabolites: A critical review. Anal. Bioanal. Chem..

[34] Guignardi Z., Schiavon M., Pilon-Smits E.A.H., Winkel L.H.E., Lin Z.-Q. (2017). Biochemistry of Plant Selenium Uptake and Metabolism. Selenium in Plants: Plant Ecophysiology.

[35] White P.J., Bowen H.C., Parmaguru P., Fritz M., Spracklen W.P., Spiby R.E., Meacham C.M., Mead A., Harriman M., Trueman J.L. (2004). Interactions between selenium and sulphur nutrition in *Arabidopsis thaliana*. J. Exp. Bot..

[36] Mobini M., Khoshgoftarmanesh A.H., Ghasemi S. (2019). Biofortification of onion bulb with selenium at different levels of sulfate. J. Plant Nutr..

[37] Kopsell D.A., Randle W.M. (1999). Selenium Affects the S-alk(en)yl Cysteine Sulfoxides among Short-day Onion Cultivars. J. Am. Soc. Hortic..

[38] Tian M., Hui M., Thannhauser T., Pan S., Li L. (2017). Selenium-Induced Toxicity Is Counteracted by Sulfur in Broccoli (*Brassica oleracea* L. var. italica). Front. Plant Sci..

[39] Põldma P., Tõnutare T., Viitak A., Luik A., Moor U. (2011). Effect of Selenium Treatment on Mineral Nutrition, Bulb Size, and Antioxidant Properties of Garlic (*Allium sativum* L.). J. Agric. Food Chem..

[40] Pérez B.M., Lipinski M.V., Filippini F.M., Chacón-Madrid K., Arruda Z.A.M., Wuilloud G.R. (2019). Selenium biofortification on garlic growth and other nutrients accumulation. Hortic. Bras..

[41] Zafeiriou I., Gasparatos D., Ioannou D., Kalderis D., Massas I. (2022). Selenium Biofortification of Lettuce Plants (*Lactuca sativa* L.) as Affected by Se Species, Se Rate, and a Biochar Co-Application in a Calcareous Soil. Agronomy.

[42] Põldma P., Moor U., Tõnutare T., Herodes K., Rebane R. (2013). Selenium treatment under field conditions affects mineral nutrition, yield and antioxidant properties of bulb onion (*Allium cepa* L.). Acta Sci. Pol. Hortorum Cultus.

[43] Republic of Serbia, Republic Hydrometeorological Service of Serbia Agricultural meteorology 2022. https://www.hidmet.gov.rs/ciril/meteorologija/agro.php.

[44] Liang B., Gao T., Zhao Q., Ma C., Chen Q., Wei Z., Li C., Li C., Ma F. (2018). Effects of Exogenous Dopamine on the Uptake, Transport, and Resorption of Apple Ionome Under Moderate Drought. Front. Plant Sci..

[45] Golubkina N.A., Folmanis G.E., Tananaev I.G. (2012). Comparative evaluation of selenium accumulation by *Allium* species after foliar application of selenium nanoparticles, sodium selenite and sodium selenate. Dokl. Biol. Sci..

[46] Zhang J.Y., Yu F.Y., Fu Z.H., Fu Y.H., Liu S.N., Chen M.L., Li Y.J., Sun Q.Z., Chang H.Q., Zhou W.L. (2019). Pathway and driving forces of selenite absorption in wheat leaf blades. Plant Soil Environ..

[47] Carignato A., Vázquez-Piqué J., Tapias R., Ruiz F., Fernández M. (2020). Variability and Plasticity in Cuticular Transpiration and Leaf Permeability Allow Differentiation of Eucalyptus Clones at an Early Age. Forests.

[48] Moravcevic D., Krstic M., Gvozdanovic-Varga J., Kostic Z.A., Vujosevic A., Kilibarda S., Vukovic S. (2022). The content of metals and metalloids in bulbs of different genotypes of *Allium* species. Book of Abstracts, Proceedings of the 11th International Symposium of Agricultural Sciences “AgroReS 2022", Trebinje, Bosnia and Herzegovina, 26–28 May 2022.

[49] Kothari D., Lee W.-D., Kim S.-K. (2020). *Allium* Flavonols: Health Benefits, Molecular Targets, and Bioavailability. Antioxidants.

[50] Skrypnik L., Feduraev P., Styran T., Golovin A., Katserov D., Nebreeva S., Maslennikov P. (2022). Biomass, Phenolic Compounds, Essential Oil Content, and Antioxidant Properties of Hyssop (*Hyssopus officinalis* L.) Grown in Hydroponics as Affected by Treatment Type and Selenium Concentration. Horticulturae.

[51] Astaneh R.K., Bolandnazar S., Nahandi F.Z., Oustan S. (2018). Effect of selenium application on phenylalanine ammonia-lyase (PAL) activity, phenol leakage and total phenolic content in garlic (*Allium sativum* L.) under NaCl stress. Inf. Process. Agric..

[52] Golubkina N., Zamana S., Seredin T., Poluboyarinov P., Sokolov S., Baranova H., Krivenkov L., Pietrantonio L., Caruso G. (2019). Effect of Selenium Biofortification and Beneficial Microorganism Inoculation on Yield, Quality and Antioxidant Properties of Shallot Bulbs. Plants.

[53] Ghasemi K., Bolandnazar S., Tabatabaei S.J., Pirdashti H., Arzanlou M., Ebrahimzadeh M.A., Fathi H. (2015). Antioxidant properties of garlic as affected by selenium and humic acid treatments. N. Z. J. Crop Hortic. Sci..

[54] Chomchan R., Siripongvutikorn S., Puttarak P. (2017). Selenium bio-fortification: An alternative to improve phytochemicals and bioactivities of plant foods. Funct. Foods Health Dis..

[55] Soil Map Serbia—Pedoloska Karta Jugoslavije. https://esdac.jrc.ec.europa.eu/content/soil-map-serbia-pedoloska-karta-jugoslavije.

[56] Singh S., Latare A., Singh S.K., Biswas D.R., Srinivasamurthy A.C., Datta P.S., Jayasree G., Jha P., Sharma K.S., Katkar N.R., Raverkar P.K., Ghosh K.A. (2019). Determination of soil reaction (pH), soluble salts (EC) and redox potential (Eh) of soil. Soil Analysis.

[57] Kogut B.M. (2012). Assessment of the humus content in arable soils of Russia. Eurasian Soil Sci..

[58] Marchetti A., Piccini C., Francaviglia R., Mabit L. (2012). Spatial Distribution of Soil Organic Matter Using Geostatistics: A Key Indicator to Assess Soil Degradation Status in Central Italy. Pedosphere.

[59] Lebedev S.A., Gunina G.N., Ashinov Y.N., Kravchenko P.N., Bedanokov M.K., Lebedev S.A., Kostianoy A.G. (2020). Ecological Conditions of Soils in the Republic of Adygea. The Republic of Adygea Environment. The Handbook of Environmental Chemistry.

[60] Pachura P., Ociepa-Kubicka A., Skowron-Grabowska B. (2016). Assessment of the availability of heavy metals to plants based on the translocation index and the bioaccumulation factor. Desalination Water Treat..

[61] Köleli N., Demir A., Kantar C., Atag G.A., Kusvuran K., Binzet R., Hakeem K.R., Sabir M., Ozturk M., Murmet A.R. (2015). Chapter 22—Heavy Metal Accumulation in Serpentine Flora of Mersin-Findikpinari (Turkey)—Role of Ethylenediamine Tetraacetic Acid in Facilitating Extraction of Nickel. Soil Remediation and Plants.

[62] Odili E.F., Njoku L.K., Soyoye A. (2018). Heavy Metals in Soils and Plants around Industries in Agbara Industrial Estate, Ogun State, Nigeria. J. Environ. Prot. Sci..

[63] Kilibarda S.N., Vuković S.Z., Milinčić D.D., Mačukanović-Jocić M.P., Jarić S., Kostić A.Ž. (2022). Phytochemical and Antioxidant Properties of *Athamanta turbith* (L.) Brot Collected from Serbia. Biol. Life Sci. Forum.

[64] RStudio Team (2022). Integrated Development for R.

